# Dexamethasone counteracts hepatic inflammation and oxidative stress in cholestatic rats *via* CAR activation

**DOI:** 10.1371/journal.pone.0204336

**Published:** 2018-09-25

**Authors:** Daniela Gabbia, Luisa Pozzo, Giorgia Zigiotto, Marco Roverso, Diana Sacchi, Arianna Dalla Pozza, Maria Carrara, Sara Bogialli, Annarosa Floreani, Maria Guido, Sara De Martin

**Affiliations:** 1 Department of Pharmaceutical and Pharmacological Sciences, University of Padova, Padova, Italy; 2 Institute of Agricultural Biology and Biotechnology, CNR, Pisa, Italy; 3 Department of Chemical Sciences, University of Padova, Padova, Italy; 4 Department of Medicine, General Pathology and Cytopathology Unit, University of Padova, Padova, Italy; 5 Department of Surgery, Oncology and Gastroenterology, University of Padova, Padova, Italy; Texas A&M University, UNITED STATES

## Abstract

Glucocorticoids (GCs) are currently used for the therapeutic management of cholestatic diseases, but their use and molecular mechanism remain controversial. The aims of this study were 1) to assess the therapeutic effect of a 2-week treatment with the GC dexamethasone on hepatic damage in bile duct-ligated rats; 2) to investigate its effect on the activation of the nuclear receptors (NRs) pregnane X receptor (PXR), constitutive androstane receptor (CAR) and GC receptor (GR), and NF-kB, as well as on oxidative stress and bile acid (BA) hepatic composition. Cholestasis was induced by ligation of bile duct (BDL animals) in 16 male Wistar-Kyoto rats, and eight of them were daily treated by oral gavage with 0.125 mg/ml/kg DEX for 14 days. Eight Sham-operated rats were used as controls. Severity of cholestasis was assessed histologically and on plasma biochemical parameters. The nuclear expression of NF-kB (p65), GR, PXR and CAR was measured in hepatic tissue by Western Blot. Oxidative stress was evaluated by measuring malondialdehyde, carbonylated proteins, GHS and ROS content in rat livers. LC-MS was used to measure the plasma and liver concentration of 7 BAs. Histological findings and a significant drop in several markers of inflammation (p65 nuclear translocation, mRNA expressions of TNF-α, IL-1β, IL-6) showed that DEX treatment reversed cholestasis-induced inflammation, and similar results have been obtained with oxidative stress markers. The nuclear expression of p65 and CAR were inversely correlated, with the latter increasing significantly after DEX treatment (p<0.01 *vs* vehicle). Hepatic BA levels tended to drop in the untreated cholestatic rats, whereas they were similar to those of healthy rats in DEX-treated animals. Plasma BAs decreased significantly in DEX-treated animals with respect to untreated cholestatic rats. In conclusion, DEX reduces inflammation and oxidative stress in BDL rats, and probably CAR is responsible for this effect. Therefore, this NR represents a promising pharmacological target for managing cholestatic and inflammatory liver diseases.

## Introduction

Glucocorticoids (GCs) are currently employed in the treatment of several liver diseases, although their use remains controversial. The well-known anti-inflammatory activity of GCs might partly account for their beneficial effect in cholestatic patients, but the risk of GCs also affecting the patient’s defences against the deleterious action of accumulated biliary components needs to be considered too. *In vivo* animal studies have already demonstrated that the expression of MRP2, a transporter responsible for the secretion of potentially toxic compounds into the bile, is upregulated by dexamethasone (DEX), a synthetic GC used to treat inflammatory processes [1–3]. It has also been reported that this effect is not due to GC Receptor (GR) activation alone, but also involves other nuclear receptors (NRs), such as the constitutive androstane receptor (CAR), whose nuclear translocation is activated by GCs [4]. CAR and the pregnane X receptor (PXR) are activated by xenobiotics and are involved in detoxification and the elimination of bile acids and drugs [5]. Mechanistic observations (reviewed by Assenat *et al*. [6]) point to a cross-talk between these NRs, and particularly to a GR-CAR/PXR-CYP2/3 signal transduction cascade. It has also been established that GR interaction with the NF-κB family of proinflammatory transcription factors leads to the latter’s functional inhibition [7]. Interference with the NF-κB pathway is considered the main mechanism of action of GCs, which are widely prescribed as anti-inflammatory and immunosuppressant drugs, and for the pharmacological management of the chronic liver inflammation caused by cholestatic diseases. Evidence suggests that the molecular mechanism behind these effects involves both an increase in the expression of IκBa [8], which prevents the nuclear translocation of NF-κB, and a direct interaction with its p65 subunit in the nucleus [9,10]. It has also been demonstrated, in primary human hepatocytes [11] and in experiments *in vivo* with mice [12] that proinflammatory cytokines, such as interleukins 6 and 1β, decrease the expression of both CAR and PXR and, concomitantly, of several cytochrome P450 (CYP) isoforms and other drug metabolizing systems transcriptionally controlled by these receptors [13]. In particular, these NRs are known to control the enzymes of the CYP3A subfamily (CYP3A4 in humans, CYP3A1 and CYP3A2 in rats) responsible for metabolizing more than 50% of currently- used medications [14]. We recently demonstrated that CYP3A-mediated drug metabolism is differently affected by early- and late-stage cholestasis and, on these grounds, we suggested that PXR and CAR might be targeted therapeutically to promote CYP3A-mediated liver detoxification [15]. Cholestasis is a bile flow impairment that leads to insufficient amounts of bile in the duodenum [16]. Cholestatic liver diseases can be caused by genetic defects, mechanical obstructions, toxins, or immune system dysregulation. They give rise to an altered bile composition and liver tissue damage. The retention and accumulation of toxic hydrophobic bile acids (BA) in the hepatocytes lead to cell toxicity by inducing apoptosis [17,18]. This BA- induced apoptosis was found to be the result of oxidative stress in human colon adenocarcinoma cells [19]. It was also recently demonstrated that administering ursodeoxycholic acid (UDCA)—one of the leading pharmacological interventions for cholestatic disease—inhibits the proliferation of colon cancer cells by regulating oxidative stress [20].

In light of these considerations, the aims of the present study were 1) to assess the therapeutic effect of DEX on hepatic damage in a well-established and reproducible rat model of BDL-induced cholestatic disease [15]; 2) to investigate its mechanism of action in depth by analyzing its effect on hepatic NRs and NF-κB activation, oxidative stress and BA composition; and 3) to ascertain whether DEX treatment affects CYP3A expression in BDL rats.

## Materials and methods

### Reagents

Dimethyl sulfoxide (DMSO), 40% acrylamide solution, sodium chloride, sodium dodecyl sulfate (SDS), skimmed milk powder, Tween 20, perchloric acid (PCA), thiobarbituric acid (TBA), trichloracetic acid (TCA), 1,1,3,3-tetramethoxypropane (TEP), dinitrophenyl hydrazine (DNPH), HCl, glutathione (GSH), KH_2_PO_4,_ o–phthaldehyde, methanol, ethanol, and ethyl acetate were purchased from Sigma-Aldrich Italy (Milan, Italy). Sucrose, Tris and magnesium chloride were obtained from Applichem (Chicago, Illinois, USA) and Complete Protease Inhibitor Cocktail from Roche (Milan, Italy). Ultrapure-grade water was produced with a Pure-Lab Option Q apparatus (Elga Lab Water, High Wycombe, UK). Mouse anti-CYP3A1, rabbit anti-CYP3A2, rabbit polyclonal anti-PXR, rabbit polyclonal anti-CAR, mouse monoclonal anti-GR and HRP-conjugated anti-mouse antibodies were purchased from Abcam (Cambridge, UK). Mouse monoclonal anti-p65, rabbit polyclonal anti IκBα, rabbit polyclonal anti-calnexin, rabbit polyclonal anti-GAPDH and mouse monoclonal anti-HDAC1 antibodies were obtained from Santa Cruz Biotechnology (Santa Cruz, CA, USA). HRP-conjugated anti-rabbit antibody was obtained from Millipore (Billerica, MA, USA), and HRP-conjugated anti-goat antibody from Jackson Immuno Research (West Grove, PA, USA). Cholic acid (CA), deoxycholic acid (DCA), chenodeoxycholic acid (CDCA), glycocholic acid (GCA), glycochenodeoxycholic acid (GCDCA), taurocholic acid (TCA), taurodeoxycholic acid (TDCA), taurochenodeoxycholic acid (TCDCA) and cholic acid-2,2,3,4,4-d5 (CA-d5—used as an internal standard–IS) were purchased from Sigma-Aldrich (St. Louis, MO, USA). All standard stock solutions were prepared by dissolving each bile acid in the appropriate amount of methanol to obtain individual stock solutions at 1 mg/mL.

### Animal model and treatment

The procedures involving animals were conducted in accordance with national and international regulations (Directive 2010/63/EU), and suitable measures were taken to minimize pain or discomfort. All procedures were reviewed and approved by the Ethics Committee of the University of Padova (OPBA), and by the Italian Ministry for the care and use of laboratory animals [Prot. no. 24, 2015] and fulfil the criteria described in the ARRIVE guidelines (S1 Fig). In this study, 24 male Wistar Kyoto rats (Charles River, Boston, MA) were divided into two experimental groups: one group (Healthy) consisted of Sham-operated animals (n = 8), whereas cholestasis was induced in the other (n = 16) by BDL and resection, as reported elsewhere [15]. One week after surgery, this latter group was divided into two subgroups: 8 rats (DEX) were treated daily by oral gavage with 0.125mg/ml/kg of DEX in corn seed oil (vehicle) for 14 days, while 8 (Vehicle) were treated with vehicle for the same period. After 14 days, the rats were sacrificed under isoflurane anesthesia, blood samples were collected by intracardiac puncture and the liver was promptly removed. A portion of each liver was kept in 10% neutral formalin for histological examination and the remainder was washed in buffer containing 50 mM TRIS, 150 mM KCl, 2mM EDTA (pH 7.4), then frozen in liquid nitrogen and stored at -80°C.

### Assessment of liver function and immunohistochemical study

Severity of cholestasis was assessed from histological examination of liver sections, and serum concentrations of albumin, alkaline phosphatase (ALKP) and total and conjugated bilirubin. Standard histological techniques were used to process liver samples. Sections obtained from paraffin-embedded liver tissues were stained with hematoxylin and eosin (H&E), and Masson trichrome for collagen. The following antibodies were used in this study to evaluate liver damage by immunohistochemistry: cytokeratin 7 (Cell-Marque, Germany, dilution 1:200), as a marker of ductular reaction of the damaged liver areas, and alpha-smooth muscle actin (ASMA, Cell Marque; Rocklin, CA, USA; dilution 1:100), as a marker of hepatic stellate cells activation. The immunohistochemical study was performed as described in Guido *et al*. [21]. The same pathologist, blinded to the study groups of origin of the liver sections, conducted all histological examinations.

### Quantification of CYP3A gene expression by qRT-PCR

RNA was obtained from 150 mg of liver tissue after purification with a commercial RNA isolation kit (Promega Corporation, Madison, WI), according to the manufacturer’s instructions, as described in detail previously [15]. qRT-PCR analysis was performed to measure CYP3A1 and CYP3A2 gene expression using a one-step commercial kit purchased from Takara (Mountain View, CA, USA), as reported elsewhere [14]. All samples were run in triplicate. To calculate the relative mRNA expression according to the ΔΔCt method [22], the cycle threshold (Ct) values were determined and β-actin was used as the housekeeping gene. The primers used in this study are reported in Table 1.

**Table 1:** Primer sequences used in the study, NCBI reference sequences, and amplicon sizes (base pairs).

Gene	Forward primer	Reverse primer	RefSeq	Size (bp)
**CYP3A1**	5’- cca-tca-cgg-aca-cag-aaa-tg—3’	5’–ctt-tcc-cca-taa-tcc-cca-ct—3’	**NM013105**	102
**CYP3A2**	5’–agt-ggg-gat-tat-ggg-gaa-ag—3’	5’–caa-tga-tgg-gga-aca-tct-cc—3’	**NM153312**	119
**CYP2B1**	5’–aac gga ttc agg agg aag cc—3’	5’–gcg ctc tcc aaa caca at gg—3’	**NM001134844.1**	131
**IL-1β**	5’–aaa-tgc-ctc-gtg-ctg-tct-ga- 3’	5’–caa-ggc-cac-agg-gat-ttt-gtc- 3’	**NM031512.2**	135
**IL-6**	5’–aag-cca-gag-tca-ttc-aga-gca-a- 3’	5’–ggt-cct-tag-cca-ctc-ctt-ct- 3’	**NM012589.2**	149
**TNFα**	5’–gat-cgg-tcc-caa-caa-gga-gg- 3’	5’–gct-tgg-tgg-ttt-gct-acg-ac- 3’	**X66539.1**	138
**β-ACTIN**	5’–gcc-acc-agt-tcg-cca-tgg-a—3’	5’–ttc-tga-ccc-ata-ccc-acc-at—3’	**NM031144**	163

### Preparation of the nuclear and microsomal fractions of liver tissue

Nuclear and cytosolic fractions were obtained according to Gabbia *et al*. [15]. After homogenization and centrifugation, the nuclear fraction was isolated at 9000 g for 30 minutes, then collected and stored at -80°C. Microsomal fractions were obtained as previously reported [14,23] and stored at -80°C. The protein content of these fractions was assessed with a commercially-available kit (Thermo Fisher BCA Protein Assay kit), using a bovine serum albumin standard calibration curve. The purity of nuclear and cytosolic extracts was assessed by incubating Western Blot membranes with anti-GAPDH and anti-HDAC1, respectively (Data not shown).

### Western blot analyses

Western blot analyses were run to measure IκB expression, CYP protein expression and NR expression in the nuclear fraction using 30 μg per lane of cytosolic, microsomal or nuclear fraction, as described elsewhere [15]. The following primary antibodies were used: anti-GR (dilution 1:1000), anti-CAR (1:500), anti-PXR (1:500), anti-p65 (1:250), anti-HDAC1 (1:250), anti-CYP3A1 (1:4000), anti CYP3A2 (1:2000) and anti-calnexin (1:700). The signal intensity of the immunoreactive bands was normalized to that of GAPDH, calnexin or HDAC1 bands, for cytosolic, microsomal and nuclear fractions, respectively. The full blot images used in this study are listed in S2 Fig.

### Malondialdehyde (MDA) level

Malondialdehyde (MDA) concentrations in the liver samples were measured according to Seljeskog *et al*. [24], with some adaptations. One hundred μL of homogenate sample was mixed with perchloric acid (PCA) (0.1125 N, 300 μL) and thiobarbituric acid (TBA) (40 mM, 300 μL), shaken vigorously for 10 s, and then placed in boiling water bath for 60 min. After cooling in a freezer to -20°C for 20 min, methanol (600 μL) and 20% TCA (200 μL) were added to the suspension and mixed for 10 s. The samples were centrifuged at 10,000 rpm for 6 min, and the MDA concentration in the supernatant was quantified with a fluorimetric detector (λ_ex_ = 525, λ_em_ = 560). A standard curve was obtained from hydrolyzed 1,1,3,3-tetramethoxypropane (TEP) dissolved in water and diluted to concentrations of 33.5, 16.8, 8.4, 4.20, 2.10, 1.05 and 0.52 μM, and blank. MDA concentrations were calculated as nmol/g tissue.

### Carbonylated protein quantification

Protein oxidation levels were ascertained using the carbonyl protein assay according to Mercier *et al*. [25] and Terevinto *et al*. [26], with slight modifications. The rat liver samples were homogenized, centrifuged and incubated with dinitrophenyl hydrazine (DNPH) 0.02M in HCl 2M. After the addition of TCA 20% and centrifugation, the pellets were washed three times with ethanol and ethyl acetate (1:1), then dissolved in guanidine HCl 6M in KH_2_PO_4_ 0.02M (pH 6.5), and centrifuged. The absorbance of the supernatant was measured at 390 nm. The carbonylated protein concentration was calculated as nmol/g tissue.

### Reduced glutathione (GSH) content

GSH levels were measured as recommended by Browne and Armstrong [27], with slight modifications. The liver samples were homogenized and deproteinized with TCA 10% at 4°C for 30 min. Then 150 μl of the sample was incubated with an equal volume of o–phthaldehyde (1 mg/ml of 10% methanol) for 15 min at 37˚C. After centrifugation at 3000 rpm for 3 min, fluorescence was measured using wavelengths of excitation and emission of 350 and 420 nm, respectively. A calibration curve was obtained with a commercial standard GSH (0.78–50 μM), and GSH concentrations were calculated as μmol/g tissue.

### Reactive oxygen species (ROS) quantification in liver tissue

The concentration of ROS in liver tissue was quantified using the method described by Niknahad *et al*. [28], with some modifications. Briefly, 200 mg of liver tissue were homogenized in 2 mL of ice-cold Tris-HCl buffer (40 mM, pH  =  7.4). Tissue homogenates (100 μL) were incubated for 40 min at 37 °C with 1 mL of a solution of 2′,7′-dichlorofluorescein diacetate (10 μM) diluited 1:200 in Tris-HCl buffer. As control of tissue autofluorescence, 100 μL of tissue homogenate were incubated with 1 mL of Tris-HCl buffer in the same conditions. The fluorescence intensity of the samples was assessed using a VictorX3 multiplate reader (λex  =  485 nm and λem  =  525 nm).

### Immunofluorescence coupled with confocal microscopy

The effect of DEX on the expression and nuclear localization of CAR were evaluated *in vitro* in a human cell line of hepatoblastoma cells (HepG2), according to the protocol described in detail in Castellani *et al*. [29]. Briefly, HepG2 cells were seeded in a 24-well plate with glass coverslips. After 24 hours, cells were pre-treated either with 0.2 or 2 μM DEX for 4 hours and then with 1 ng/mL LPS for 24 hours. The effect of DEX was evaluated using untreated and LPS-treated cells as negative and positive controls, respectively. At the end of the treatment, cells were fixed in 4% paraformaldehyde and incubated with a rabbit polyclonal primary antibody antiCAR (1:100 dilution, Abcam, Cambridge, UK) for 1 h, using an Alexa Fluor 488 anti-rabbit as secondary antibody (1:500 dilution, Jackson ImmunoResearch Europe Ltd., Ely, UK). For nuclear staining, cells were incubated with DAPI (100 pg/mL, Life Technologies, Monza, Italy), after treatment with a 2 mg/mL RNAse solution (Sigma-Aldrich, Milan, Italy). The images of the immunostained cells were acquired by means of a confocal microscope Zeiss LSM 800, using a 63X magnification. ImageJ software was used to quantify the intensity of the fluorescent signal and the co-localization between the nuclear marker DAPI and CAR signal.

### Bile acid extraction and LC-MS/MS analysis

Bile acids were extracted from liver samples and plasma using the procedure reported by Yang *et al*. [30]. Liver tissues were homogenized in physiological buffer (500 μL of solution per 100 mg of tissue). For each tissue sample, the homogenate was divided into four 100 μL aliquots. Using the standard addition method, each aliquot was spiked with 10 μL of a mixed standard solution containing the BAs of interest at final concentrations of 0, 10, 20 and 50 μM, respectively, and with 2.5 μM of IS. The obtained samples were added with 1 mL of ice-cold alkaline acetonitrile, vortexed and centrifuged at 14800 rpm at 4°C for 10 min. Supernatants were collected and dried under nitrogen flow. Before the injection into the LC-MS/MS system, sample were reconstituted with 100 μL of methanol and deionized water (85:15 v:v). As far as plasma is concerned, the protocol was the same, starting from ice-cold alkaline acetonitrile addition.

The LC-MS/MS system was equipped with an Ultimate 3000 UHPLC chromatograph coupled with a QExactive hybrid quadrupole-Orbitrap mass spectrometer (Thermo Fisher Scientific, Waltham, Massachusetts, USA). A Kinetex 2.6 μm EVO C18, 100 A, 100x2.1 mm (Phenomenex, Bologna, Italy) column thermostated at 25°C was used at flow rate 0.25 mL/min. Water (A) and acetonitrile (B) both 0.1% formic acid were used as eluents, with the following gradient (t, minutes): t_0-20_ 0–100% B; t_20-30_ 100% B; t_30-31_ 0% B and the seven minutes for the re-equilibrium. The MS conditions were set as follows: electrospray (ESI) ionization in negative mode with lock mass at *m/z* 112.9856, resolution 35000 in MS and 17500 in MS/MS (at *m/z* 200), AGC target 3ˑ10^6^ and 2ˑ10^5^ in MS and tandem MS, respectively; max injection time 200 ms, scan range 50–750 a.m.u, isolation window 4.0 *m/z*, normalized collision energy 60 V. The capillary voltage was 2.8 kV, capillary temperature was 320°C, auxiliary gas was nitrogen at 40 a.u. Calibration was performed with a standard solution from Thermo Fisher Scientific (Pierce ESI Negative Ion Calibration Solution). The MS data were analyzed with the Xcalibur 4.0 software (Thermo Fisher Scientific).

Each BA was quantified using the standard addition method with a four-point curve, assayed in duplicate. The peak area ratios (A/A_IS_) related to the [M-H]^-^ extracted ion chromatogram (mass accuracy 10 ppm) of the selected BA and IS were plotted. Linearity was assessed using least squares regression and showed an R^2^ >0.98 for all the considered analytes.

### Statistical analyses

GraphPad Prism 5.0 software was used (GraphPad Software Inc., San Diego, CA, USA). Data were compared using one-way ANOVA. In the event of significant differences (α = 0.05), the ANOVA was followed up with the Dunnett or Newman-Keuls *post-hoc* test. A P value <0.05 was considered statistically significant. Unless otherwise stated, data are presented as mean ± SD.

## Results

### Histology, immunohistochemistry and biochemical analyses of rat livers

**Fig 1:**
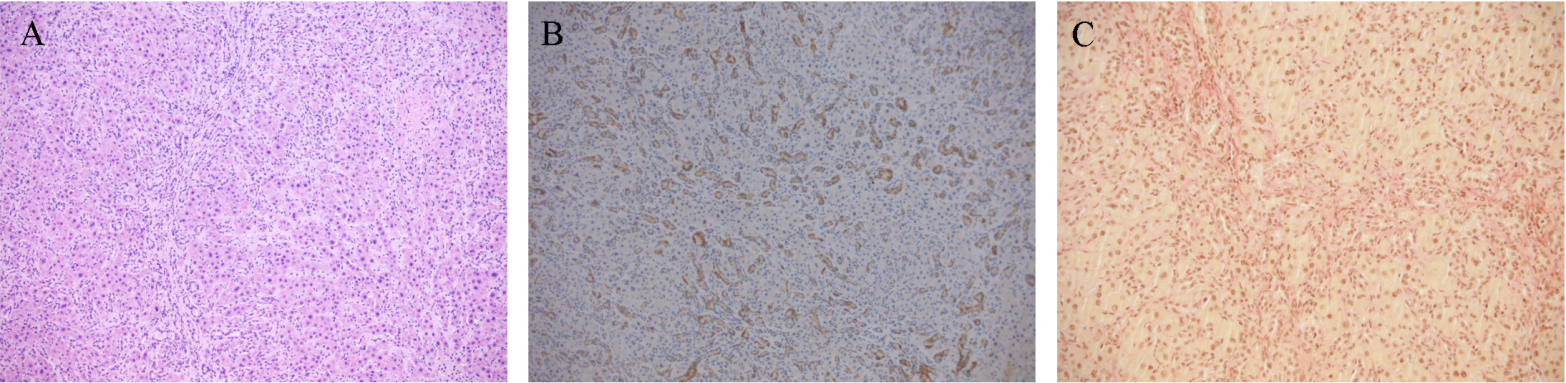
Liver histology of cholestatic rats. Representative photomicrographs of rat liver tissue from cholestatic untreated rats on H&E staining (A) ductular reaction is highlighetd by CK7 immunostain (B) and is associated with collagen deposition as shown by Van Gieson stain (C).

**Fig 2:**
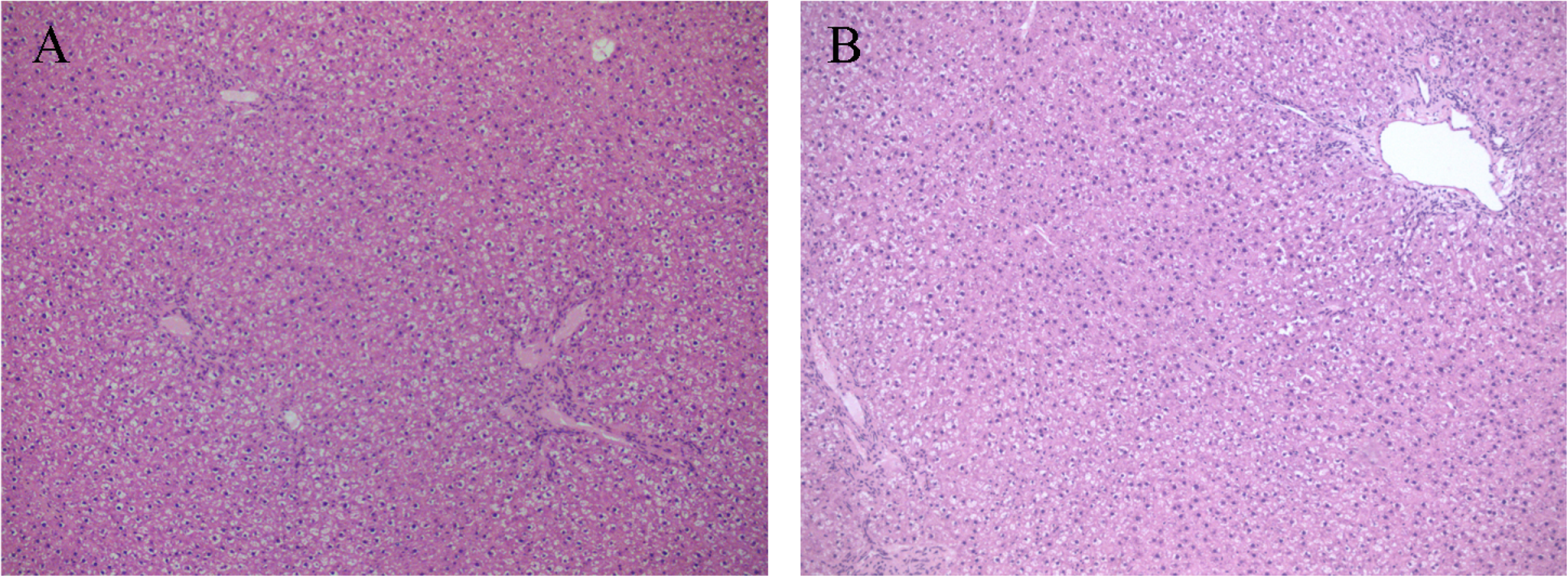
Liver histology of healthy and DEX-treated rats. Representative photomicrographs of rat liver tissue from healthy (A) and BDL rats treated with DEX (B) stained with H&E.

**Fig 3:**
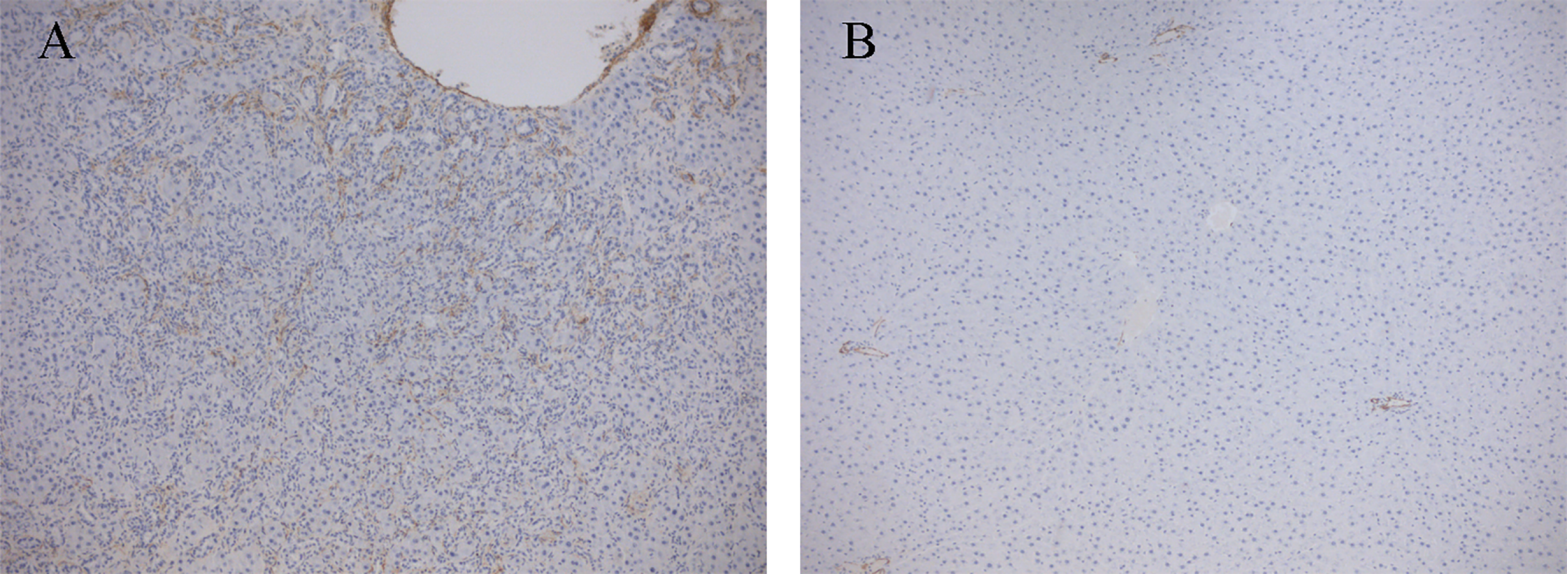
Activation of hepatic stellate cells. Representative photomicrographs of rat liver tissue from untreated (A) and DEX-treated (B) BDL rats stained with ASMA, a marker of HSCs activation.

Cholestasis was induced by ligating the common bile duct [15]. After sacrificing the animals, liver function was assessed on the basis of liver histology (Fig 1), and biochemical tests (albumin, ALT, AST, ALKP, and serum total and conjugated bilirubin), as shown in Table 2. Histopathological examination showed portal and lobular inflammation (Fig 1A) and severe ductular reaction, evidenced by cytokeratin 7 positivity (Fig 1B) replacing most lobular parenchyma and associated with collagen deposition (Fig 1C). The liver samples obtained from the healthy and the BDL rats treated with DEX were indistinguishable (Fig 2A and 2B). Immunostaining with alpha-smooth muscle actin (αSMA), used as a marker of activated hepatic stellate cells (HSCs), was positive in the damaged areas of the liver of all the cholestatic rats treated with vehicle (Fig 3A) and in none of DEX-treated animals (Fig 3B).

**Table 2:** Biochemical parameters of liver function in healthy and cholestatic rats.

	Healthy	Cholestatic	
		Vehicle	DEX
**Albumin (g/L)**	37.2 ± 2.2	30.7 ± 4.3	40.5 ± 4.3*^,^^##^
**ALKP (U/L)**	184.7± 15.3	340.7 ± 73.3*	247.6 ± 117.8
**ALT (U/L)**	41.8 ± 14.3	149.7 ± 39.1***	61.2 ± 25.8 ^###^
**AST (U/L)**	83.8 ± 7.5	570.7 ± 104.3***	230.5 ± 193^##^
**Total bilirubin (mmol/L)**	1.0 ± 0.2	204.0 ± 15.0***	66.7 ± 22.4^###^

Results are given as mean ± SD.

*P < 0.05,

***P < 0.001 *vs* healthy animals;

^##^P < 0.01,

^###^P < 0.001 *vs* cholestatic rats treated with vehicle.

### Effect of DEX treatment on NR activation

**Fig 4:**
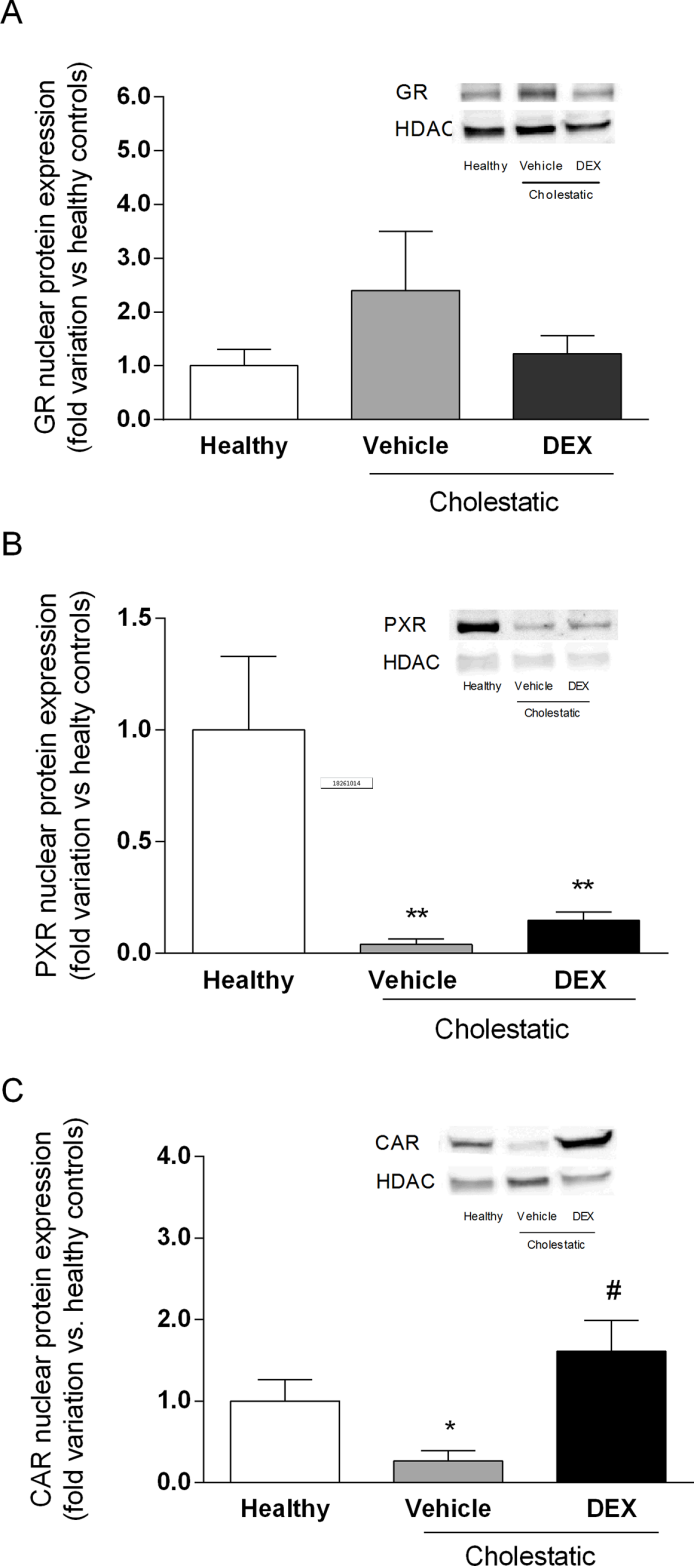
NR activation in rat livers. Densitometric analysis of the immunoreactive bands of: (A) GR nuclear protein expression; (B) PXR nuclear protein expression; and (C) CAR nuclear protein expression. A representative Western blot experiment showing NR nuclear protein expression is shown on the right of each graph. HDAC was used as loading control. Data are expressed as mean ± SEM. ANOVA followed by the Neuman-Keuls *post-hoc* test. *P < 0.05, **P < 0.01 vs healthy rats; #P < 0.05 vs cholestatic rats treated with vehicle.

To ascertain whether cholestasis and DEX treatment modify the nuclear levels of GR, PX and CAR (the three main NRs involved in the mechanism of action of GCs), we measured their nuclear protein expression. As shown in Fig 4, there were no significant differences in GR nuclear expression between the three groups of animals (Fig 4A). The nuclear expression of PXR (Fig 4B) was significantly reduced in both the cholestatic animal groups, however, irrespective of any DEX treatment (p<0.01 vs healthy rats). The nuclear expression of CAR (Fig 4C) was found significantly reduced in the cholestatic rats treated with vehicle (p<0.05 vs healthy animals) but restored to normal by DEX treatment (p<0.05 vs vehicle).

**Fig 5:**
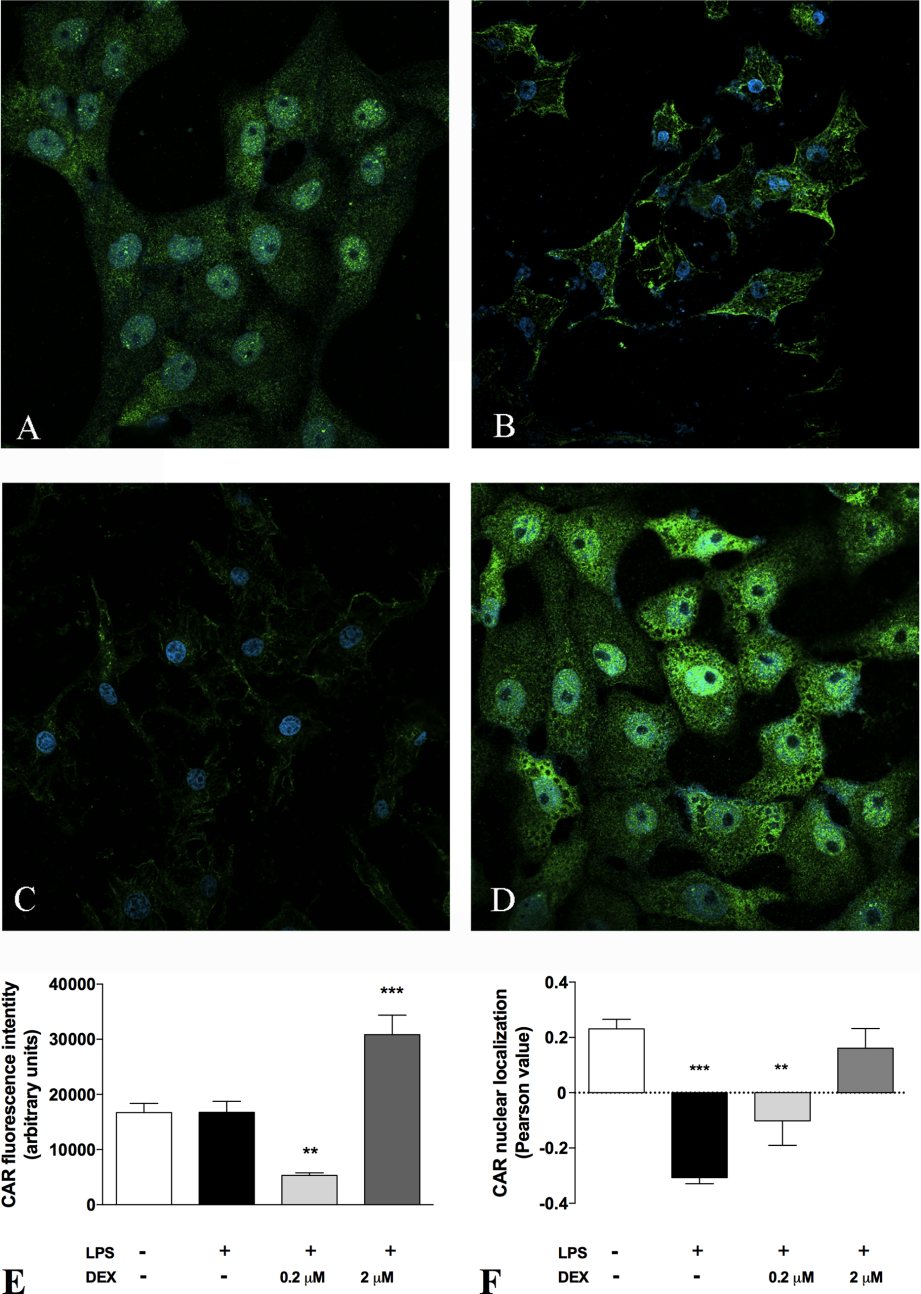
Immunofluorescence staining of CAR in HepG2 cells. Immunostaining for the CAR after incubation in basal condition (control), with 10 μg/ml LPS for 24h, 0.2 μM DEX + 10 μg/ml LPS for 24h and 2 μM DEX + 10 μg/ml LPS for 24h. Values are means ± S.E.M. (n = 10). The histograms report the intensity of fluorescence (arbitrary units). Representative images from five independent experiments are shown. Cell nuclei are stained blue with DAPI. ANOVA followed by the Dunnett post-hoc test. *P < 0.05; ***P < 0.001 vs control.

This finding was confirmed by analyzing the mRNA expression of CYP2B1, a well-known target gene of CAR [31]. As reported in S3 Fig, CYP2B1 mRNA levels resemble CAR nuclear expression in the different groups of animals, indicating that the transcriptional activity of CAR is also modulated by DEX treatment. We obtained further evidences of DEX effect on CAR activation by an in vitro experiment performed in HepG2 cells (Fig 5). We demonstrated that in these cells DEX was able to restore the constitutive activation of CAR, which was lost after a detrimental stimulus, such as LPS treatment, in a dose-dependent manner, as indicated by the Pearson value (Fig 5F), which gives an index of the co-localization between the signals of CAR and the nuclear marker DAPI. Furthermore, in HepG2 cells DEX caused changes in CAR expression, since the green fluorescence was significantly increased after 24 hours of incubation (Fig 5E). Taken together, these results concur in demonstrating that DEX leads to CAR activation and increase of transcriptional activity.

### Effect of DEX treatment on hepatic inflammation

**Fig 6:**
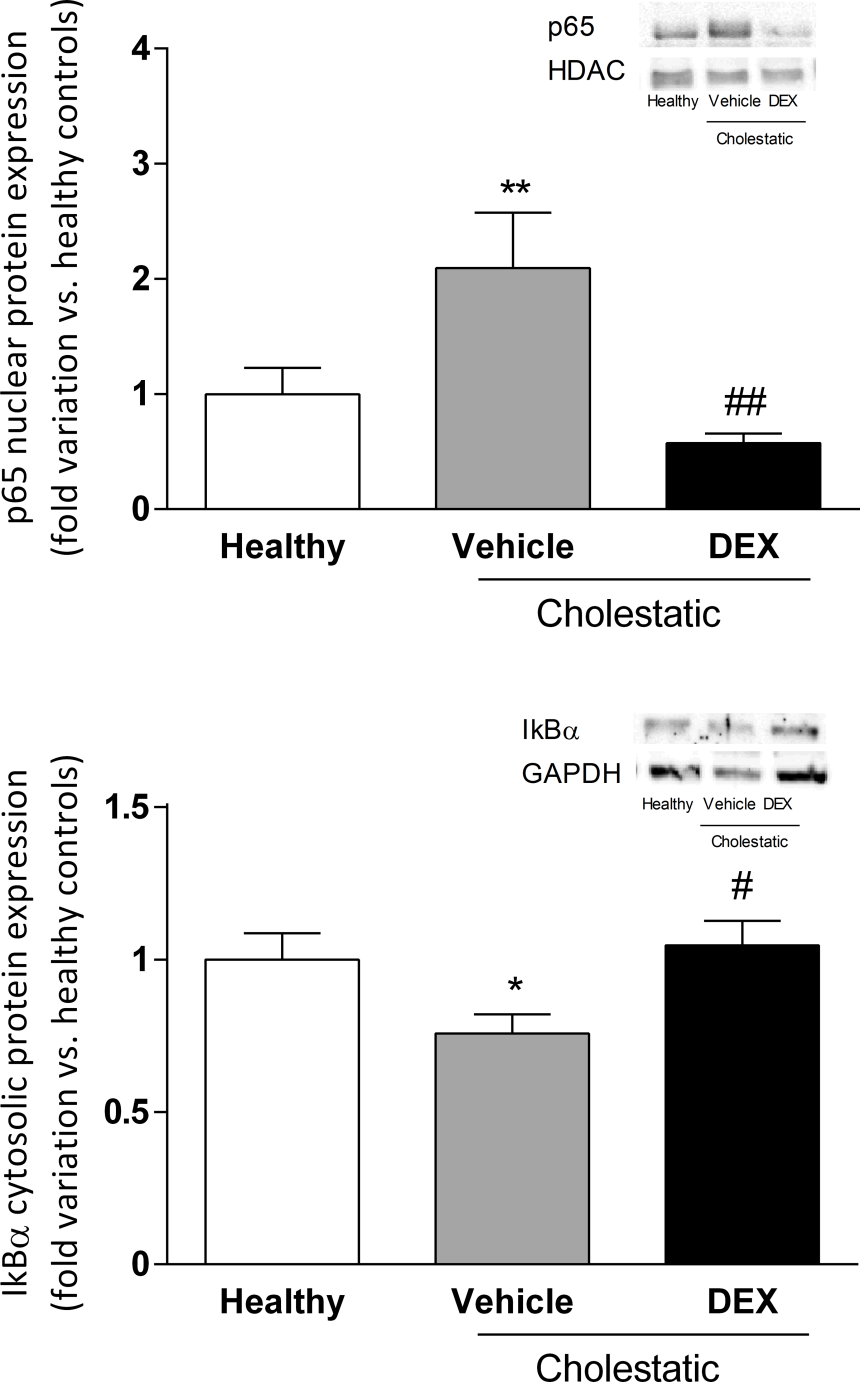
**A: p65 nuclear protein expression in rat livers.** A representative Western blot experiment showing p65 nuclear protein expression is shown on the right of the graph. Data are expressed as mean ± SEM. **B: IκBαcytosolic protein expression in rat livers.** A representative Western blot experiment showing IκBαprotein expression in the cytosolic fraction is shown on the right of the graph. Data are expressed as mean ± SEM. ANOVA followed by the Neuman-Keuls post-hoc test. *P<0.05, **P < 0.01 vs healthy rats; ^#^ P<0.05, ^##^P < 0.01 vs cholestatic rats treated with vehicle.

To see whether DEX (0.125 mg/kg/ml) had an anti-inflammatory effect on the liver of cholestatic rats, we measured the nuclear expression of p65, a NF-κB subunit known to be kept inactive in the cytosol by inhibitors, such as IκBα, that mask the NF-κB nuclear localization sequence, and to translocate into the cell nucleus in response to inflammatory stimuli [29]. As expected, Fig 6A shows that p65 nuclear expression was significantly higher (p<0.01) in cholestatic rats treated with vehicle than in healthy animals, and DEX treatment counteracted this cholestasis-related inflammation, since p65 nuclear expression was significantly lower in DEX-treated rats (p<0.01 *vs* cholestatic rats treated with vehicle). Accordingly, IκBαexpression decreased significantly in the cytosolic compartment in BDL untreated animals (p<0.05) and was restored to normal levels in DEX-treated rats (Fig 6B).

**Fig 7:**
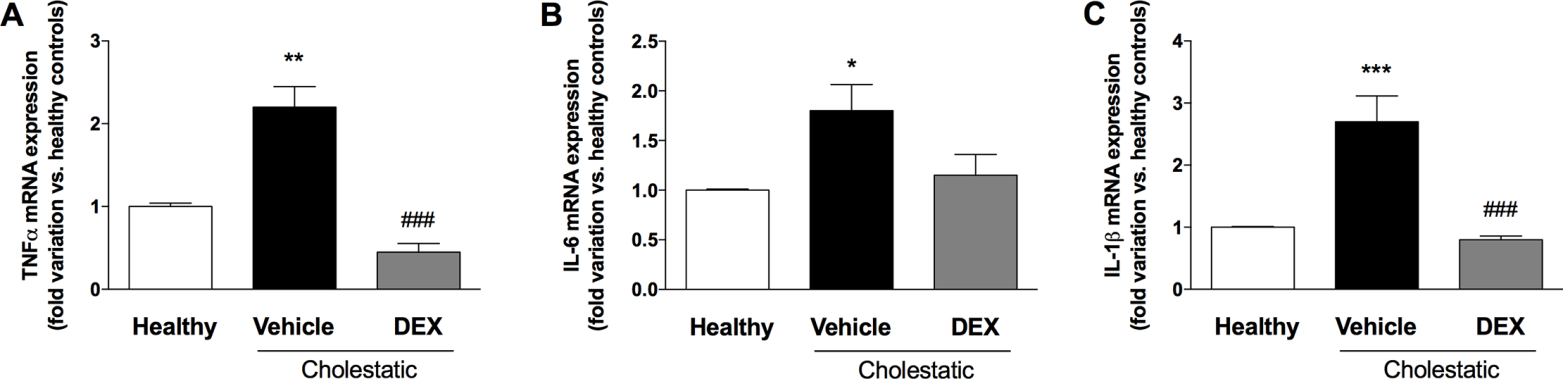
Inflammatory cytokines in rat livers. mRNA levels of (A) TNFα, (B) IL-6, and (C) IL-1β. Data are presented as means ± SEM. ANOVA followed by the Neuman-Keuls post-hoc test. *P<0.05, **P < 0.01, ***P < 0.01 vs healthy rats; ^###^P < 0.001 vs cholestatic rats treated with vehicle.

To further confirm the anti-inflammatory effect of DEX in the cholestatic liver, we analyzed the mRNA expression of three inflammatory cytokines, i.e. TNFα, IL-6 and IL-1β. In accordance with what observed regarding the activation of NF-κB, the mRNA expressions of these three cytokines increased in the livers of untreated cholestatic rats and was restored to normal levels in the BDL animals treated with DEX (Fig 7).

### Oxidative stress in rat liver

**Fig 8:**
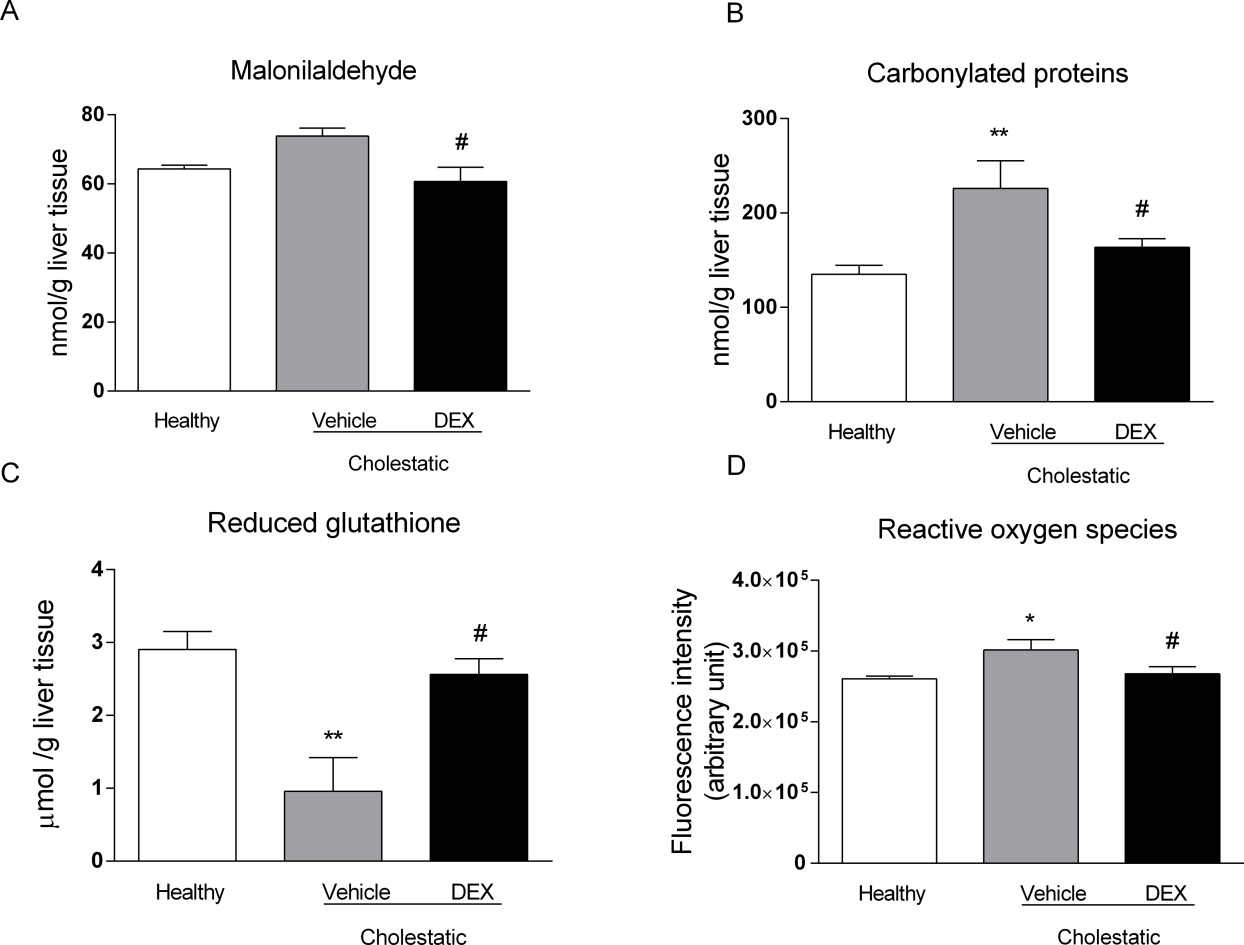
Oxidative stress in rat livers. A: malondialdehyde (MDA) levels; B: carbonylated proteins levels; C: reduced glutathione (GSH) content. D: ROS content. Data are presented as means ± SEM. ANOVA followed by the Neuman-Keuls post-hoc test. *P<0.05, **P < 0.01 vs healthy rats; #P < 0.05 vs cholestatic rats treated with vehicle.

Since cholestasis is known to prompt a significant increase in periportal oxidative stress in patients with primary sclerosing cholangitis (PBC) [32], we measured three markers of oxidative stress in rat livers: malondialdehyde (MDA), carbonylated proteins, and reduced glutathione (GSH) content. As clearly shown in Fig 8, MDA levels were significantly lower (p<0.05) in cholestatic rats treated with DEX than in those treated with vehicle (Fig 8A). Consistently with this observation, the liver content of carbonylated proteins increased significantly in the untreated cholestatic rats but returned to normal levels in their DEX-treated counterparts (Fig 8B). The level of the antioxidant agent GSH was significantly lower in the cholestatic rats treated with vehicle than in the healthy animals (p<0.01), but it was increased by DEX treatment (p<0.5 *vs* untreated cholestatic rats, Fig 8C). Accordingly, ROS levels in liver tissue significantly increased in cholestatic animals (p<0.05) and restored to normal levels in BDL rats treated with DEX (Fig 8D).

### Hepatic and plasma bile acid content

**Fig 9:**
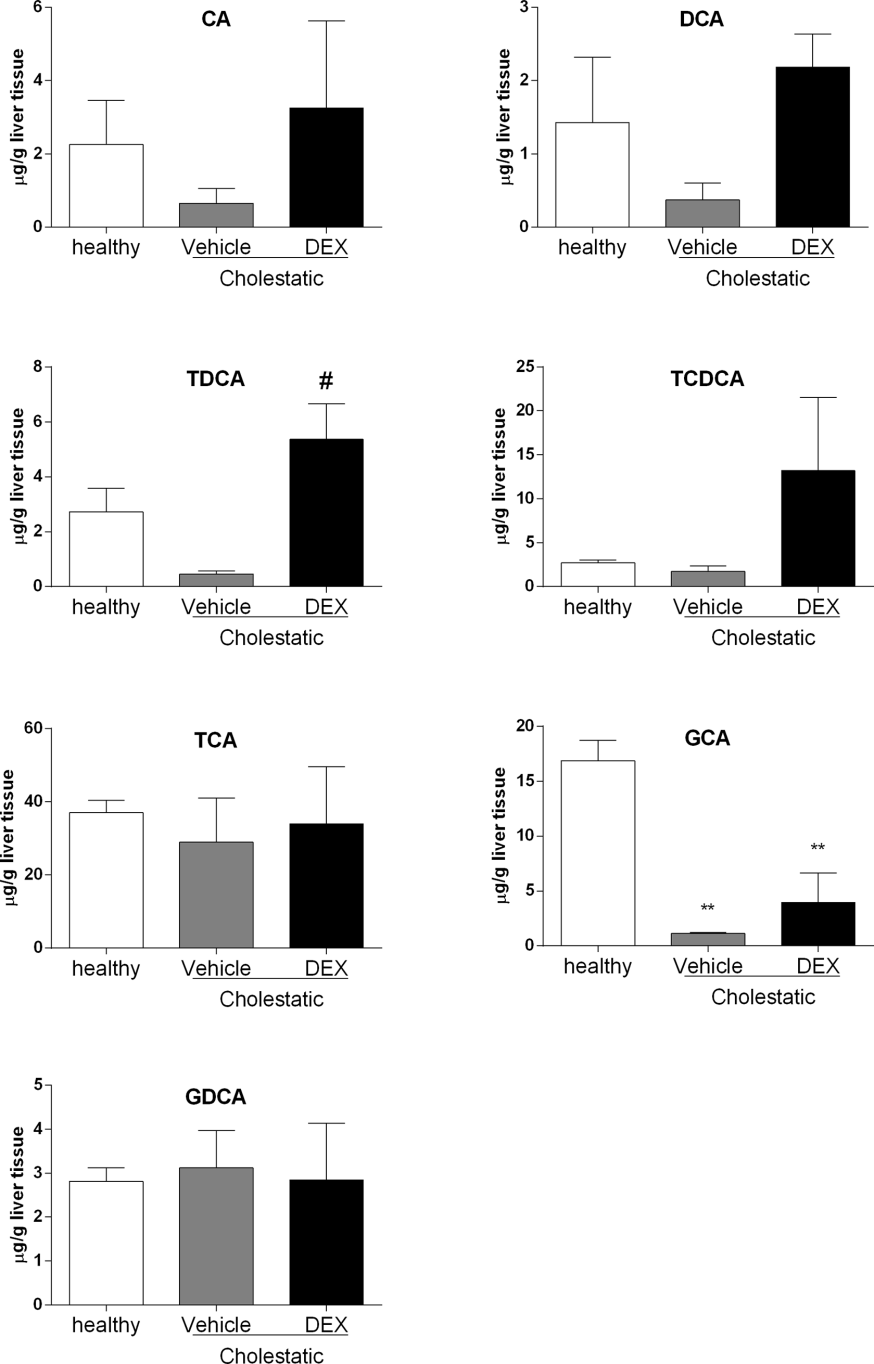
Hepatic BA levels. CA: cholic acid, DCA: deoxycholic acid, TCA: taurocholic acid, TCDCA: taurochenodeoxycholic acid, TDCA: taurodeoxycholic acid, GCA: glycocholic acid, GDCA: glycodeoxycholic acid. Data are means ± SEM. ANOVA followed by the Neuman-Keuls post-hoc test. ** P<0.01 vs healthy rats; ^#^ P<0.05 vs vehicle.

**Fig 10:**
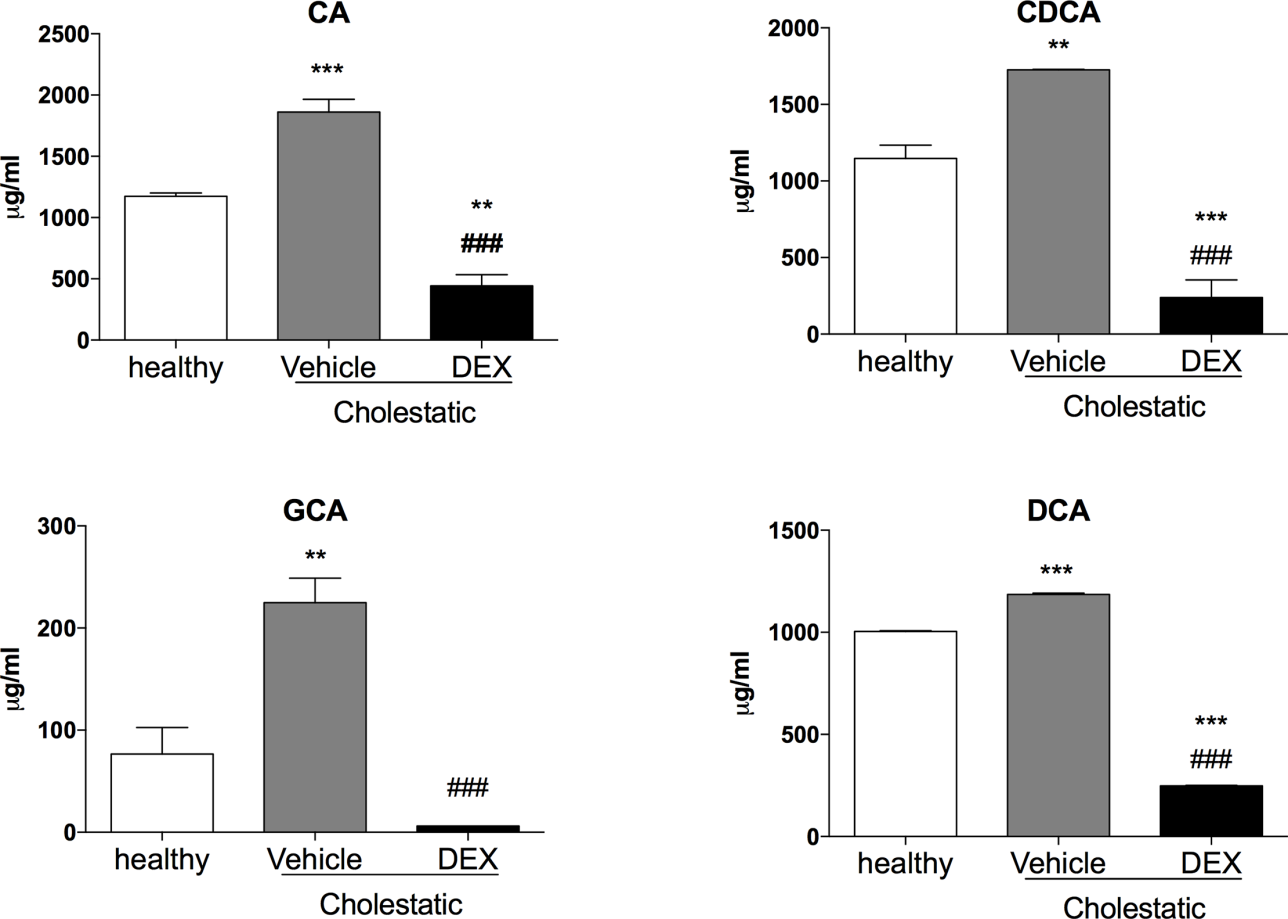
Plasma BA levels. CA: cholic acid, DCA: deoxycholic acid, GCA: glycocholic acid. Data are means ± SEM. ANOVA followed by the Neuman-Keuls post-hoc test. ** P<0.01, ***P<0.001 vs healthy rats; ^#^ P<0.05, ^###^ P<0.001 vs vehicle.

Since the ratio between different BAs is important for feedback regulation of BA synthesis and other physiological processes [33], we measured the hepatic content of the main rat BAs to ascertain whether DEX treatment affects their synthesis. As shown in Fig 9, few statistically significant differences emerged, probably due to high inter-individual variability. As far as CA, DCA and TDCA were concerned, their levels tended to drop in the untreated cholestatic animals, whereas after DEX treatment they were similar to those of healthy animals. The levels of the other BAs were similar in all three groups, with the exception of GCA, which was significantly lower in the cholestatic rats, whether they were treated with DEX or not. To further confirm the role of DEX on the amelioration of cholestatic liver injury, we measured the plasma levels of the main rat BAs. Only four BAs (CA, CDCA, GCA, DCA), as shown in Fig 10, were detectable in all the 3 groups of rats, and all of them decreased significantly in BDL rats after DEX treatment.

### CYP3A gene and protein expression

**Fig 11:**
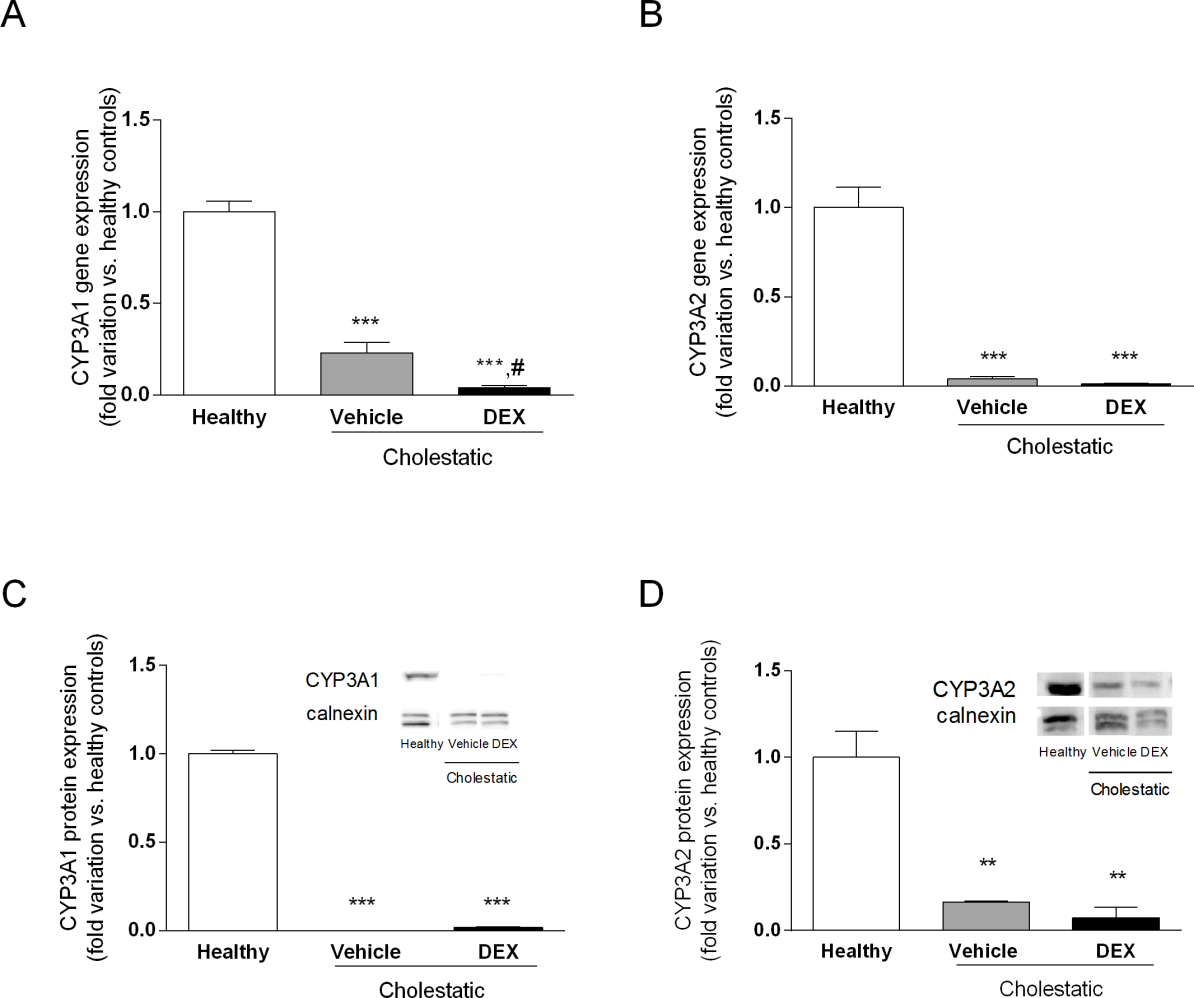
CYP3A gene and protein expression in rat livers. A: CYP3A1 gene expression. B: CYP3A2 gene expression. C: CYP3A1 protein expression in microsomal fraction. D: CYP3A2 protein expression in microsomal fraction. Western blot experiment showing NR nuclear protein expression is shown on the right of C and D graphs. Calnexin was used as loading control. Data are means ± SEM. ANOVA followed by the Neuman-Keuls post-hoc test. **P < 0.01, ***P < 0.001, vs healthy rats; ^#^P < 0.05 vs cholestatic rats treated with vehicle.

To see whether DEX treatment affects CYP3A expression in BDL rats, we measured gene and protein expressions of CYP3A1 and CYP3A2, which are the metabolically most important CYP3A isoforms in male rats. Fig 11 shows that gene and protein expressions of both enzymes were dramatically reduced in cholestasis, as previously observed [15], and that DEX treatment had no effect on this reduction.

## Discussion

Since GCs represent a therapeutic option to treat cholestatic diseases [34], we investigated in this study whether low DEX doses reduce liver inflammation and oxidative stress in a validated animal model of BDL-induced cholestasis. This model was chosen for its translational importance, since the morphological changes induced by BDL and the accumulation of BA in the rat liver resemble those of human cholestasis [35,36]. Findings can therefore be compared with those seen in patients with the two main classes of cholestatic disease, i.e. primary biliary cholangitis (PBC) and primary sclerosing cholangitis (PSC). The ability of DEX to reduce inflammation in rats is evident from our histological and biochemical findings. Having demonstrated its therapeutic efficacy, we attempted to gain further insight on the mechanisms of action of DEX: it is known to activate GR, but it also binds to other NRs in a dose-dependent manner. It is well known that DEX is a potent synthetic GC acting as a GR agonist: this NR is normally sequestered in the cytoplasm but, after GC treatment, it relocalizes to the cell nucleus, where it can bind to its DNA recognition sites, called glucocorticoid response elements (GREs), and activate the transcription of target genes [37]. In addition to this classical GC mechanism, however, when DEX is used at high doses (i.e. 100 mg/kg) [14], it has proved a very effective inducer of rat CYP3A enzymes, as a consequence of PXR activation [38]. It has also been demonstrated that DEX enhances CAR gene and protein expression in human hepatocytes [4]. These findings reveal a complex regulatory network (or cross-talk) involving different NRs, which suggests that the clinical efficacy of DEX and allied drugs may be the result of several cellular mechanisms. In this study, we measured the nuclear expression of GR, PXR, CAR and p65, a protein belonging to the NF-κB family. This family of transcription factors is involved in inflammation, a phenomenon usually involving the stimulation of toll-like receptor (TLR) 4. TLR activation leads to NF-κB translocation to the nucleus, and an increased transcription of inflammatory molecules [39]. Based on our findings, we suggest that the mechanism by which DEX attenuates inflammation (as indicated by the decrease in p65 nuclear expression and the drop of hepatic inflammatory cytokines; see Figs 6 and 7) may be CAR activation because—among the various NRs responsive to DEX—only CAR nuclear expression is significantly affected in DEX-treated rats. Further evidence of the DEX capability of activating CAR has been obtained in vitro by treating HepG2 cells with increasing concentrations of this drug (Fig 5).

Several authors have reported that exposure to high doses of GCs may raise the basal levels of reactive oxygen species (ROS) b altering the capacity for defense against oxidative damage [40], but, as far as we know, the effect of low-dose DEX on oxidative stress has not been investigated. We found that administering 0.125 mg/kg of DEX for 14 days prompted a reduction in two validated markers of oxidative stress in the liver, and an increase in the hepatic content of the antioxidant agent GSH. This result has interesting clinical implications, since several clinical studies showed that the severity of cholestasis related to changes in oxidative stress markers, for instance in intrahepatic cholestasis of pregnancy [41], and in PBC [32]. The use of antioxidant products should therefore be considered as an adjuvant therapy for cholestatic diseases. It has been shown that a combination of higher levels of reactive oxygen species (ROS) and lower levels of lithocholic acid and DCA is significantly associated with cholestasis [42]. Accordingly, we measured the liver content of the main rat BAs, to see whether DEX treatment affected their synthesis. It is worth emphasizing that, in spite of a marked variability in liver content of BAs, already observed elsewhere [42], we found a reduction in DCA in cholestatic rats that was completely reversed by DEX treatment. The same trend was apparent for CA, whereas TDCA levels were raised by DEX treatment, even vis-à-vis healthy rats. In short, our results indicate that, while low-dose DEX does not influence the liver content of all BAs, the cholestasis-induced reduction in some of them (CA, DCA and TDCA) is counteracted by DEX treatment (Fig 9). Furthermore, DEX decreased significantly the levels of 4 BAs in plasma (Fig 10), and this is accordance with the observed reduction of inflammation and oxidative stress obtained in the DEX-treated BDL-rats.

Finally, since DEX is known to be capable of activating PXR, a transcriptional regulator of the CYP3A enzymes [14], we measured the gene and protein expression of CYP3A1 and CYP3A2 (the two main CYP3A isoforms in the rat liver, and the orthologues of the human CYP3A4). Consistently with the lack of PXR activation after DEX treatment (Fig 4), we found that CYP3A enzymes were likewise not induced by this GC, but their gene and protein expression was significantly lower in cholestatic rats, with or without DEX treatment. This finding has clinical implications because we know that CYP3A enzymes (and particularly CYP3A4, in humans) can metabolize more that 50% of the drugs currently used in clinical practice. A reduction in CYP3A-mediated metabolism should therefore be expected in cholestasis, prompting changes in the pharmacokinetics of several drugs. The decrease in CYP3A enzyme levels was still apparent in BDL rats treated with DEX, although their liver function was comparable with that of healthy rats. In another model of liver disease (CCl_4_-induced liver cirrhosis), we found that only CYP3A2 transcription—not PXR expression—was reduced [14]. In line with the conclusions drawn from that earlier investigation, the present results indicate that no general conclusions can be drawn from studying a given animal model of liver disease, given that different results emerged for CCl_4_-treated and BDL rats. In the present study, we used a chronic treatment with low-dose DEX that mimics the use in humans of rifampicin, which can be administered to cholestatic patients [43]. This was done because rifampicin and DEX are the prototypical PXR-inducers in humans [44,45], and rodents [38], respectively. The dose of DEX used in this work was unable to activate neither GR or PXR, while it did unexpectedly activate only CAR.

In conclusion, this study demonstrated that low-dose DEX can reduce cholestasis-induced liver dysfunction, inflammation and oxidative stress. This effect is probably mediated by CAR activation and does not involve CYP3A-mediated metabolism. Taken together, these findings point to CAR as a promising pharmacological target for the treatment of cholestatic diseases.

## Supporting information

S1 Fig NC3Rs ARRIVE guidelines checklist. (DOCX)

S2 Fig Uncropped and un-modified blot images used in this study. (DOCX)

S3 Fig mRNA expression of CYP2B1, a target gene of CAR. ANOVA followed by the Neuman-Keuls post-hoc test. ***P<0.001 vs healthy rats; ^###^P<0.001 vs cholestatic rats treated with vehicle.
(TIFF)
