# Supplementary material for: Dexamethasone counteracts hepatic inflammation and oxidative stress in cholestatic rats via CAR activation
Source: PLoS One. 2018 Sep 25;13(9):e0204336. doi: 10.1371/journal.pone.0204336 (PMC6155538; doi:10.1371/journal.pone.0204336)

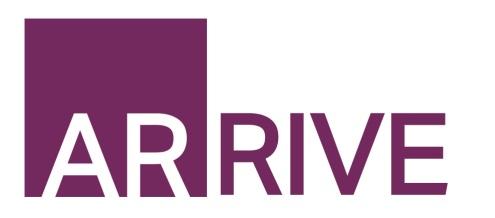


The ARRIVE Guidelines Checklist

Animal Research: Reporting In Vivo Experiments

Carol Kilkenny^1^, William J Browne^2^, Innes C Cuthill^3^, Michael Emerson^4^ and Douglas G Altman^5^

*^1^The National Centre for the Replacement, Refinement and Reduction of Animals in Research, London, UK, ^2^School of Veterinary Science, University of Bristol, Bristol, UK, ^3^School of Biological Sciences, University of Bristol, Bristol, UK, ^4^National Heart and Lung Institute, Imperial College London, UK, ^5^Centre for Statistics in Medicine, University of Oxford, Oxford, UK.*

|  | | ITEM | RECOMMENDATION | Section/ Paragraph |
| --- | --- | --- | --- | --- |
|  | 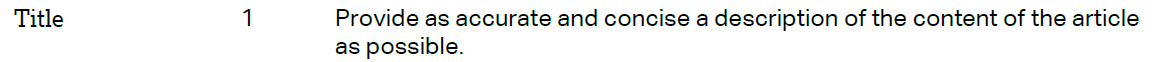 | | | Title |
|  | 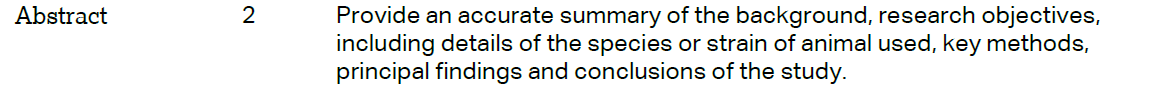 | | | Abstract |
|  | INTRODUCTION | | |  |
|  | 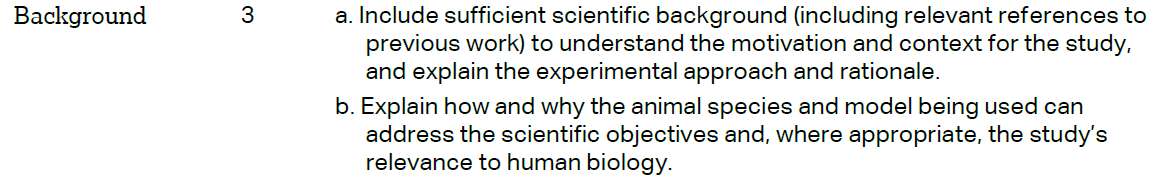 | | | Paragraph 1,  discussion paragraph 1 |
|  | 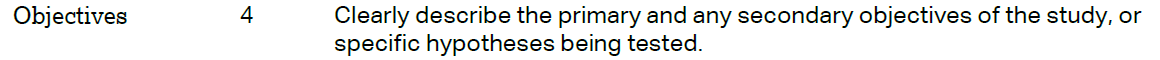 | | | Paragraph 2 |
|  | METHODS | | |  |
|  | 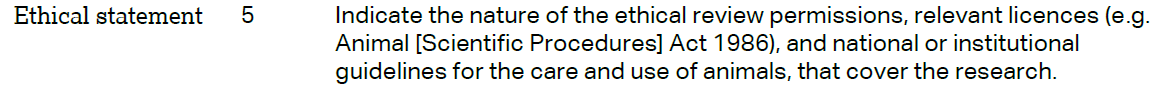 | | | Methods Paragraph 2 |
|  | 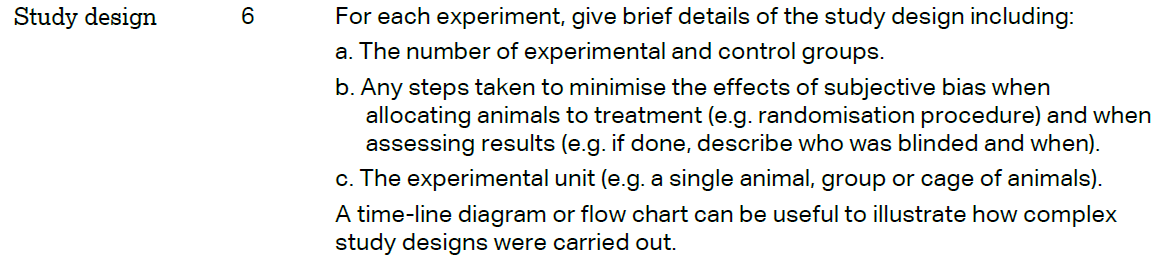 | | | Methods  Paragraph 2 and Table 2 |
|  | 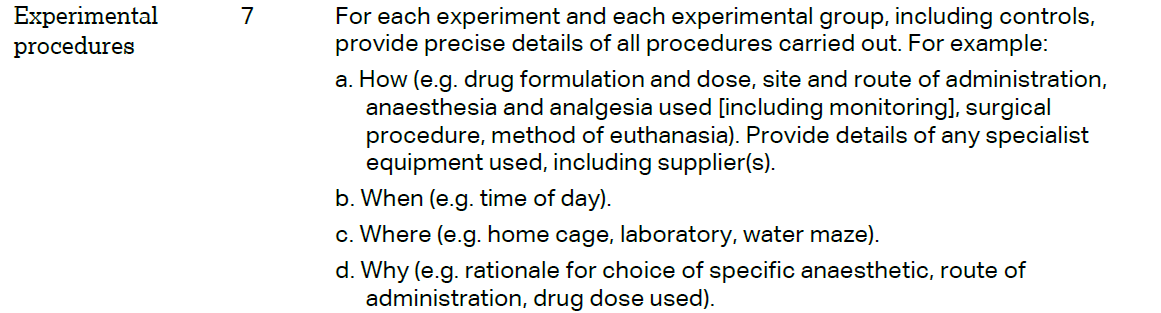 | | | Methods  Paragraph 2 |
|  | 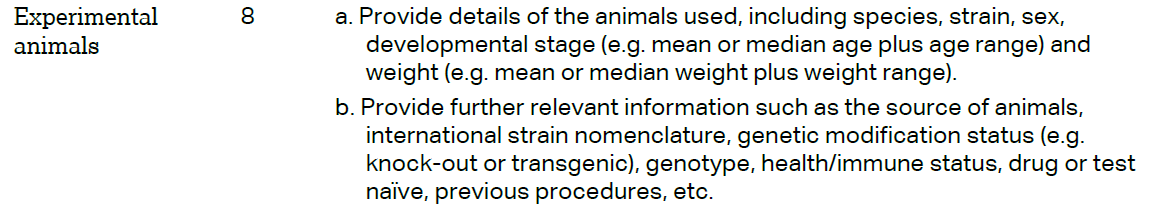 | | | Methods Paragraph 2 |

|  | 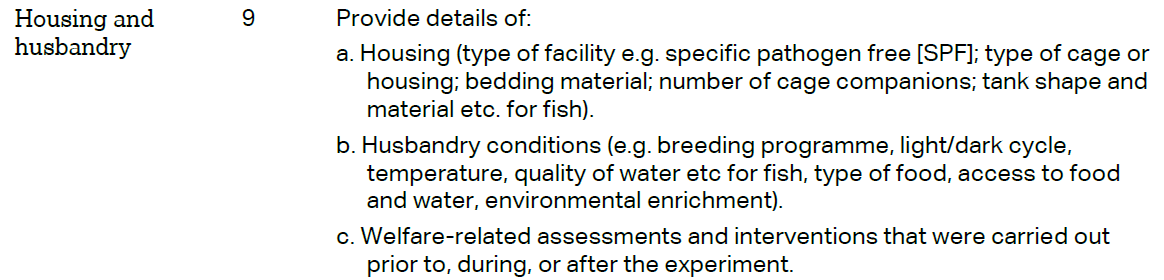 | Methods Paragraph 2 | |
| --- | --- | --- | --- |
|  | 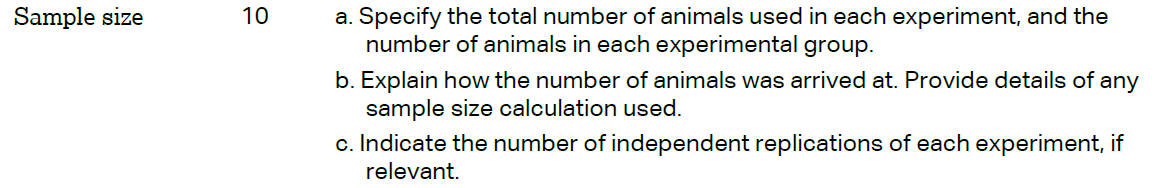 | Methods Paragraph 2 | |
|  | 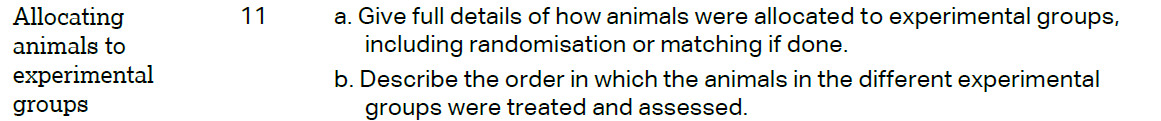 | Methods Paragraph 2 | |
|  | 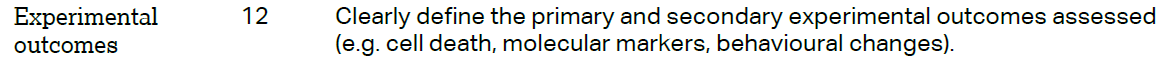 | Methods  Paragraph 3-11 and Results 1 | |
|  | 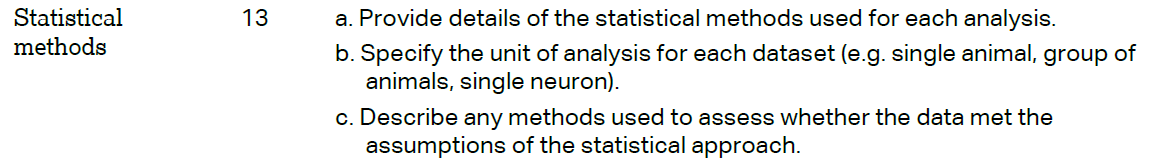 | Paragraph 12 | |
|  | RESULTS |  | |
|  | 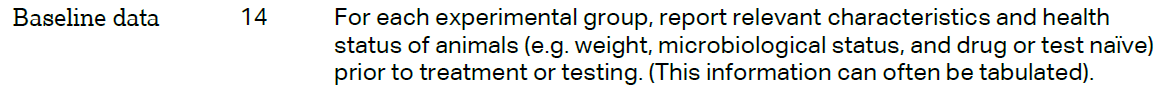 | Table 2  Result Paragraph 1 | |
|  | 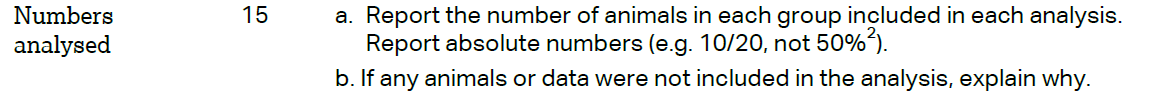 | Methods  Paragraph 2 | |
|  | 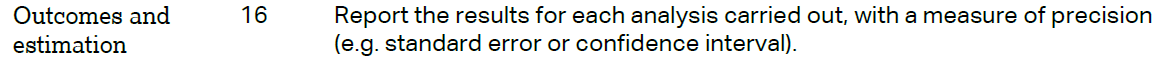 | Figures 1-6 | |
|  | 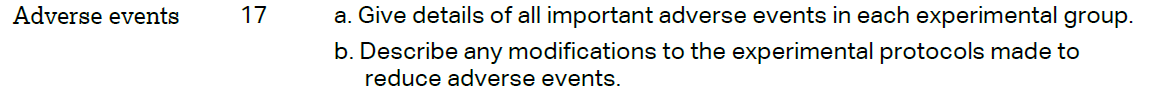 | Methods Paragraph 2 | |
|  | DISCUSSION |  | |
|  | 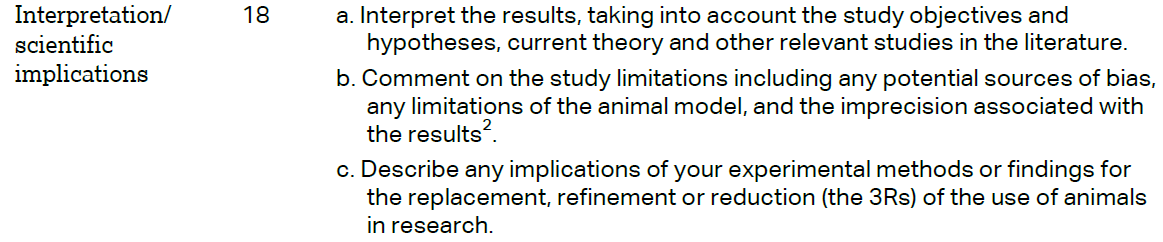 | Discussion paragraph 1-3 | |
|  | 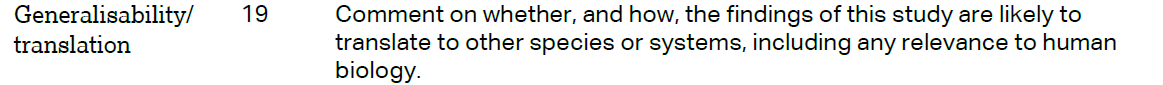 | Discussion paragraph 1-3 | |
| 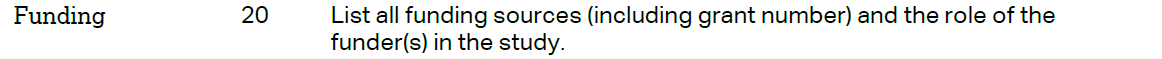 | | ParagraphFunding sources |  |


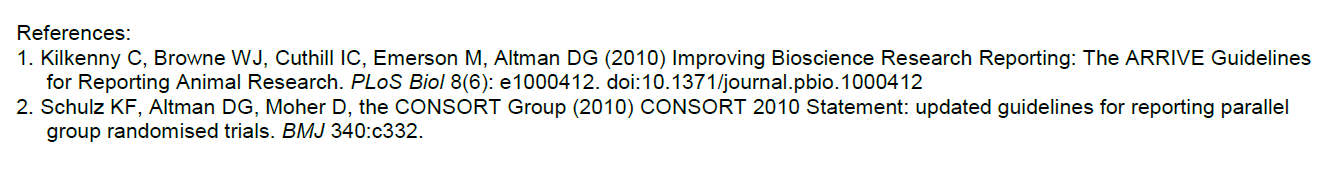

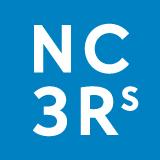

Supplement: S1 Fig — (DOCX) [file pone.0204336.s001.docx]
