# Supplementary figures and images for: Dexamethasone counteracts hepatic inflammation and oxidative stress in cholestatic rats via CAR activation
Source: PLoS One. 2018 Sep 25;13(9):e0204336. doi: 10.1371/journal.pone.0204336 (PMC6155538; doi:10.1371/journal.pone.0204336)

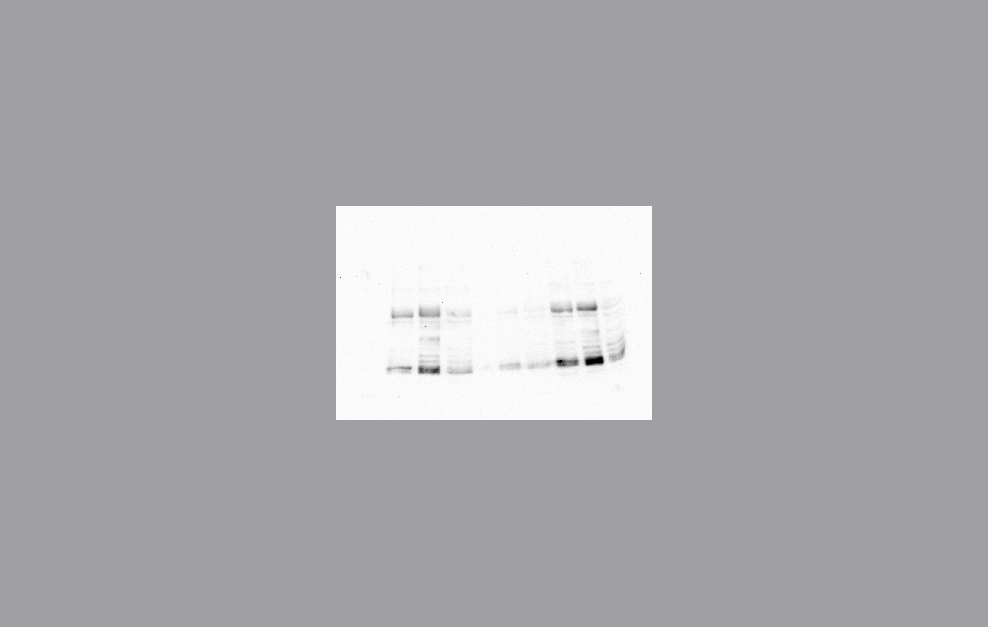
P65


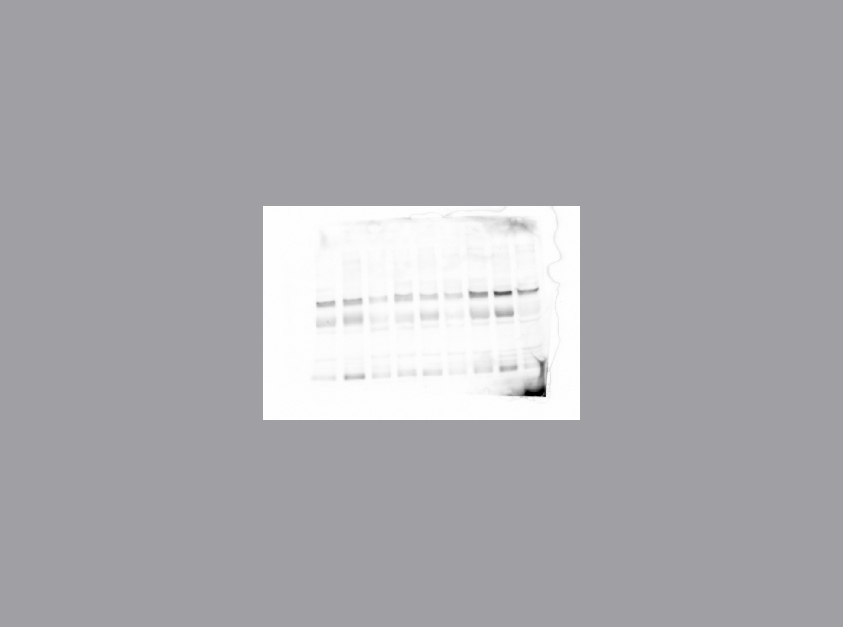
 HDAC


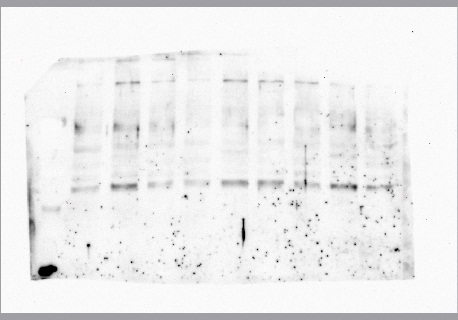
Ikb


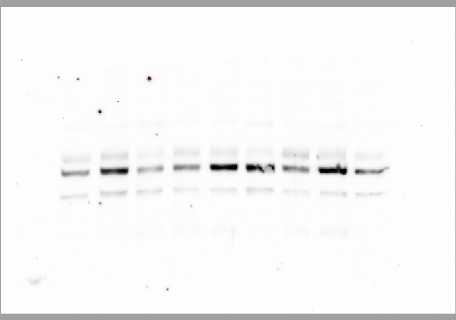
GAPDH


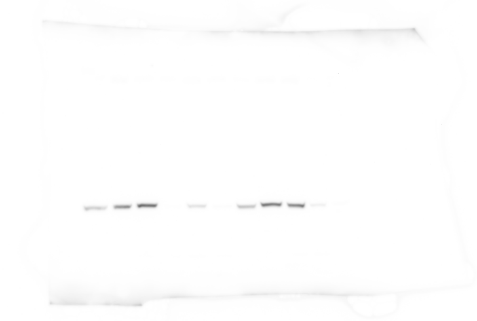
CYP3A1


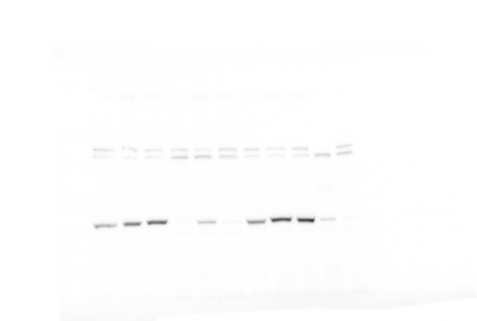
calnexin


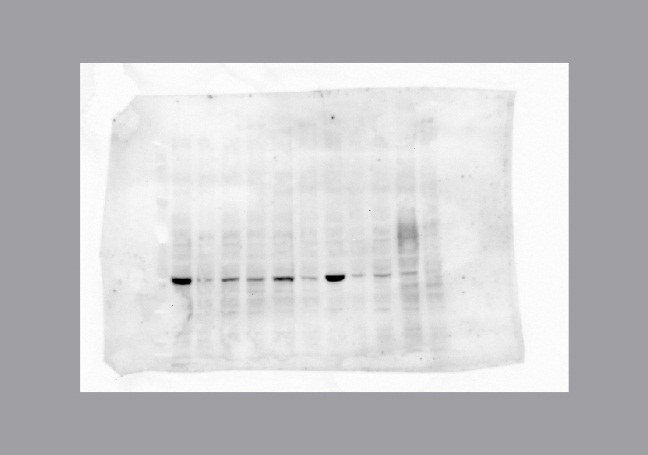
CYp3A2


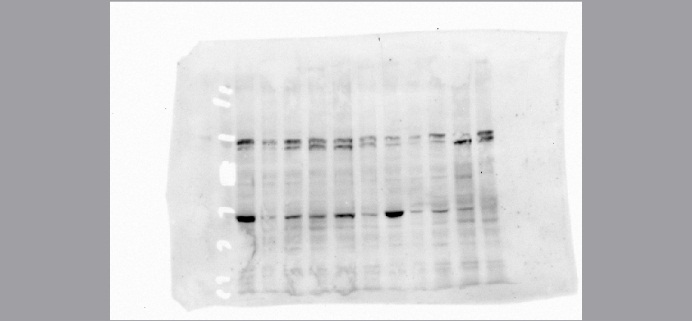
Calnexin


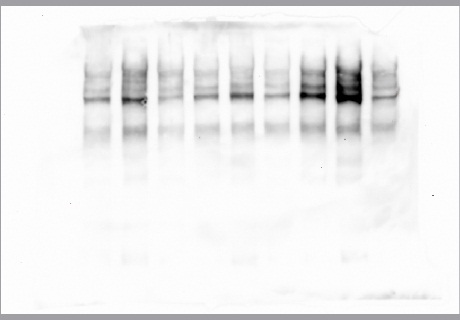
GR


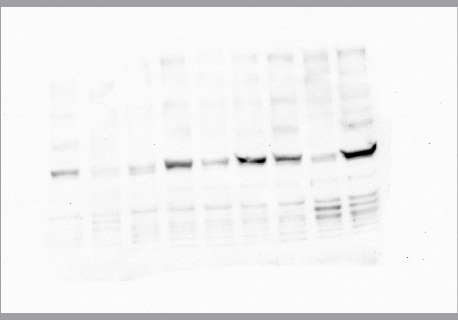
CAR


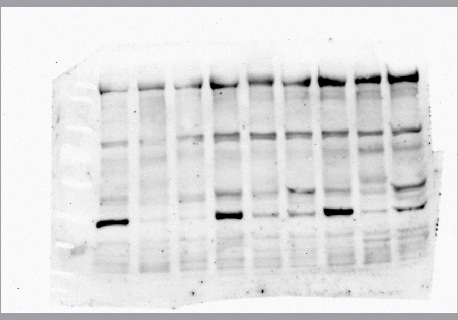
PXR


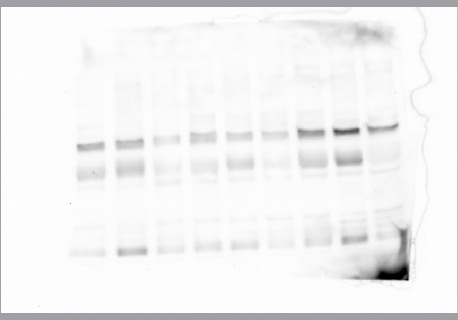
HDAC

Supplement: S2 Fig — (DOCX) [file pone.0204336.s002.docx]

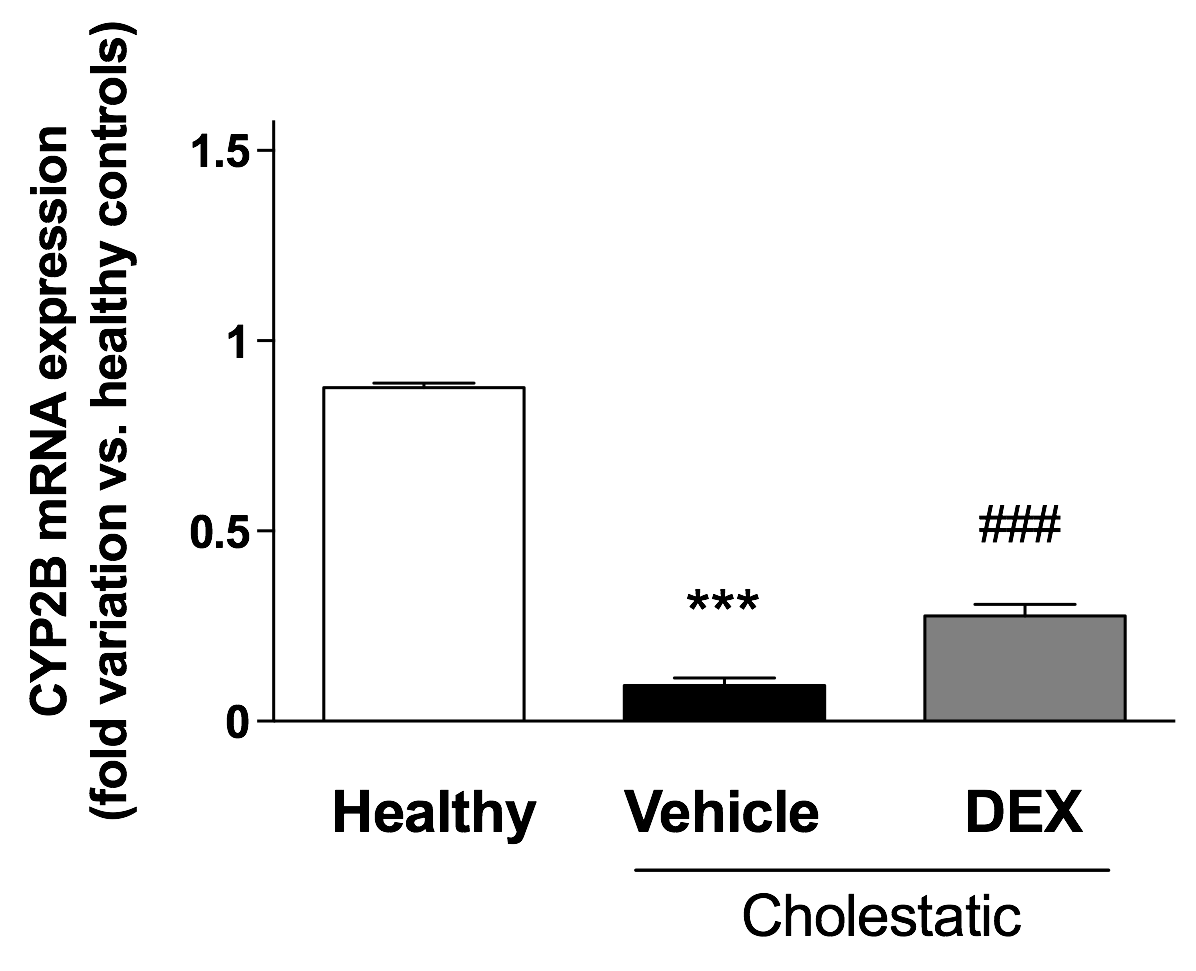

Supplement: S3 Fig — ANOVA followed by the Neuman-Keuls post-hoc test. ***P<0.001 vs healthy rats; ###P<0.001 vs cholestatic rats treated with vehicle. (TIFF) [file pone.0204336.s003.tiff]
